# Neurosarcoidosis-Induced Panhypopituitarism

**DOI:** 10.7759/cureus.43169

**Published:** 2023-08-08

**Authors:** Khalid Alfares, Hye Jeong Han

**Affiliations:** 1 Endocrinology, Diabetes, and Metabolism, King Abdulaziz University Faculty of Medicine, Jeddah, SAU; 2 Endocrinology, Diabetes, and Metabolism, Henry Ford Health System, Detroit, USA; 3 Internal Medicine, Henry Ford Health System, Detroit, USA

**Keywords:** central hypothermia, diabetes insipidus, panhypopituitarism, neurosarcoidosis, hypothalamo-pituitary sarcoidosis

## Abstract

Sarcoidosis is an inflammatory condition that can impact multiple organs in the body such as the lungs, skin, eyes, and, occasionally, the central nervous system. When sarcoidosis affects the nervous system, it is referred to as neurosarcoidosis and is estimated to occur in approximately 5%‐15% of sarcoid patients. When neurosarcoidosis affects the pituitary gland, it can result in panhypopituitarism, which can be life-threatening.

A 35-year-old male with a known diagnosis of sarcoidosis by skin biopsies presented to the hospital with altered mental status, hypernatremia, hypotension, and hypothermia. He reported symptoms of polyuria and polydipsia for several weeks before admission. Laboratory workup revealed elevated serum sodium at 167 mmol/L, high serum osmolality at 381 mOsm/kg, and low urine osmolality at 381 mOsm/kg, consistent with diabetes insipidus. Anterior pituitary hormone profile workup revealed low 8 am serum cortisol (1.9 mcg/dL) and inappropriately normal adrenocorticotropic hormone (ACTH) (34 pg/ml), low serum free testosterone (<2.5 ng/dL), low luteinizing hormone (0.7 mIU/ml), low follicular stimulating hormone (< 2.6 mIU/ml), low free T4 at 0.4 ng/dL. and inappropriately normal thyroid-stimulating hormone (TSH) at 2.77 uIU/mL. Serum prolactin was mildly elevated at 86.8 ng/mL. Angiotensin-converting enzyme level was within the normal range at 33 U/L. A diagnosis of panhypopituitarism was made. Brain MRI revealed a 3 cm mass in the suprasellar region involving the hypothalamus and bilateral optic tracts with a mass effect on the anterior third ventricle. No discrete pituitary or stalk lesion was identified. A ventriculostomy tube was placed for developing hydrocephalus. A biopsy of the suprasellar mass revealed non-caseating granuloma, confirming neurosarcoidosis. Treatment was initiated with high-dose IV corticosteroids to manage secondary adrenal insufficiency and neurosarcoidosis. He was also started on IV desmopressin and IV levothyroxine to manage his diabetes insipidus and central hypothyroidism. He was transitioned to oral therapy upon discharge.

Panhypopituitarism secondary to neurosarcoidosis is a rare presentation that can occur due to the infiltration of the pituitary gland or the infiltration of the hypothalamus affecting the hypothalamic-pituitary axis. Neurosarcoidosis should be considered a differential when evaluating patients with symptoms consistent with panhypopituitarism. Prompt diagnosis and initiation of corticosteroids and deficient hormones can be lifesaving.

## Introduction

Panhypopituitarism occurs when the pituitary gland fails to produce an adequate amount of all the pituitary gland hormones, leading to imbalances and disruptions in several physiological processes. Sarcoidosis is a condition that triggers inflammation in various organs of the body, including the lung, eyes, skin, and occasionally the central nervous system. When sarcoidosis affects the nervous system, it is referred to as neurosarcoidosis, estimated to occur in approximately 5%‐15% of patients [1]. When neurosarcoidosis affects the pituitary gland, it can destroy or compress the gland, interfering with its hormone-producing capabilities. As a result, multiple hormone deficiencies can occur, which can be life-threatening. We hereby present a rare case of neurosarcoidosis causing panhypopituitarism.

## Case presentation

A 35-year-old male with a known diagnosis of sarcoidosis eight months prior by skin biopsies presented to the hospital with altered mental status, hypernatremia, hypotension, and hypothermia. He reported symptoms of polyuria and polydipsia for several weeks before admission. Laboratory workup revealed elevated serum sodium at 168 mmol/L, high serum osmolality at 381 mOsm/kg, and low urine osmolality at 381 mOsm/kg, consistent with diabetes insipidus. Anterior pituitary hormone profile workup revealed low 8 am serum cortisol (1.9 mcg/dL) and inappropriately normal adrenocorticotropic hormone (ACTH) (34 pg/ml), low serum free testosterone (<2.5 ng/dL), low luteinizing hormone (0.7 mIU/ml), low follicular stimulating hormone (< 2.6 mIU/ml), low free T4 at 0.4 ng/dL, and inappropriately normal thyroid-stimulating hormone (TSH) at 2.77 uIU/mL. Serum prolactin was mildly elevated at 86.8 ng/mL. Angiotensin-converting enzyme level was within the normal range at 33 U/L (Table 1). A diagnosis of panhypopituitarism was made.

**Table 1:** Lab results ACTH: adrenocorticotropic hormone

	Patient's lab	Normal range
Serum Na	168 mmol/L	275-295 mOsm/kg
Serum osmolality	398 mOsm/kg	275-295 mOsm/kg
Urine osmolality	381 mOsm/kg	300-1100 mOsm/kg
8 am serum cortisol	1.9 mcg/dL	6.0-18.4 mcg/dL
Serum ACTH	34 pg/ml	7.2-63 pg/ml
Serum free testosterone	<2.5 ng/dL	249-836 ng/dL
Luteinizing hormone	0.7 mIU/ml	1.7-8.6 mIU/ml
Follicular stimulating hormone	<2.6 mIU/ml	1.5-12.4 mIU/ml
Free T4	0.4 ng/dL	0.8-1.7 ng/dL
Thyroid-stimulating hormone	2.77 ulU/ml	0.27-4.2 mIU/ml
Serum prolactin	86.8 ng/mL	4.0-15.2 ng/ml

**Figure 1:**
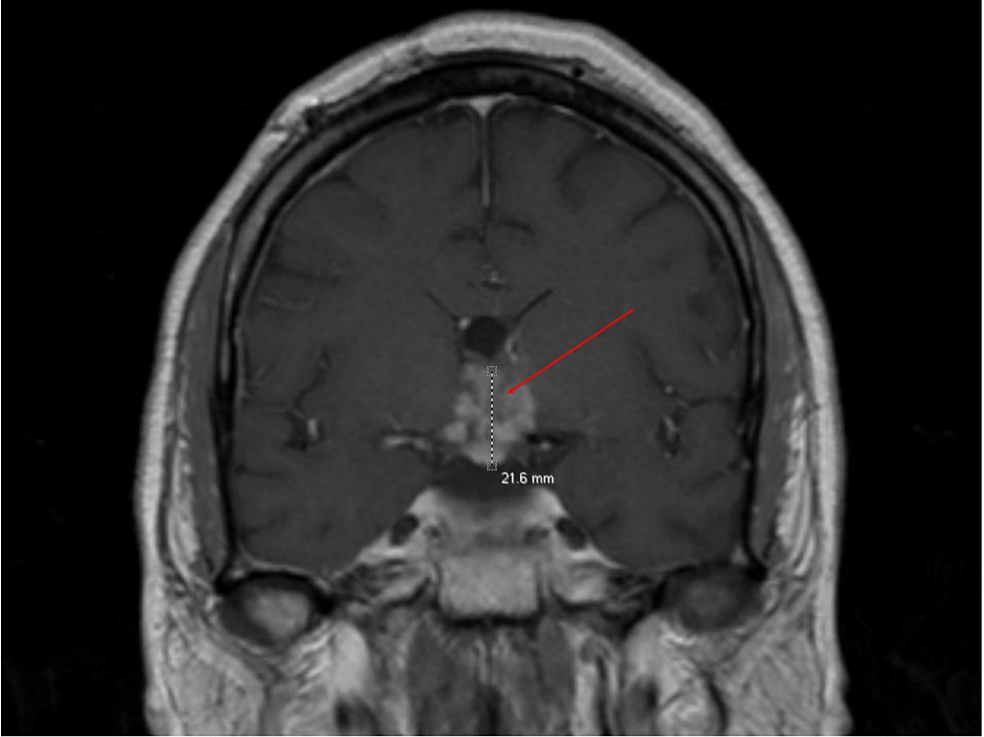
Coronal T1 post-contrast MRI pituitary showing a multilobulated compressing mass within the inferior third ventricle, hypothalamus, and optic chiasm

Brain MRI revealed a 3 cm mass in the suprasellar region involving the hypothalamus, optic chiasm, and optic tracts with a mass effect on the anterior third ventricle. No discrete pituitary or stalk lesion was identified (Figure 1).

A ventriculostomy was done to treat hydrocephalus that developed due to the mass extending into the third ventricle. An endoscopic transsphenoidal biopsy of the suprasellar mass revealed non-caseating granuloma, confirming neurosarcoidosis. Treatment was initiated with high-dose IV corticosteroids to manage secondary adrenal insufficiency and neurosarcoidosis. He was also started on IV desmopressin and IV levothyroxine to manage diabetes insipidus and central hypothyroidism. His sodium levels normalized. He was transitioned to oral therapy and deemed stable for discharge with the plan to schedule a follow-up appointment and reevaluate his pituitary hormones outpatient.

## Discussion

The symptoms of neurosarcoidosis vary depending on the specific anatomic structure affected by sarcoid lesions [2]. It has been observed that a small percentage, approximately 5-10% of patients diagnosed with sarcoidosis exhibit neurological symptoms. However, neurosarcoidosis comprises only 1% of all sarcoidosis cases [3]. Initial assumptions were made based on post-mortem findings from patients with hypothalamic-pituitary failure that suggested sarcoid infiltration of either the hypothalamus or pituitary gland could result in partial or total tissue destruction [4]. Recent research has revealed that panhypopituitarism is primarily caused by hypothalamus infiltration, which offers valuable insight into the underlying mechanisms of the condition [5]. Occasionally, it may mimic a pituitary mass, but it is rare [6]. In this situation, it may be necessary to undergo a biopsy and obtain histological confirmation. Endocrine disturbances can occur in 2-26% of cases. These disturbances can lead to various conditions such as diabetes insipidus, galactorrhoea, and amenorrhoea. However, as is panhypopituitarism, impaired thermoregulation is much less frequent [3,5]. Our patients had features of both.

When it comes to identifying sellar lesions, there is a vast array of possible diagnoses to consider. This can include pituitary tumors, craniopharyngiomas, germ cell tumors, meningiomas, cysts of Rathke's pouch, Arachnoid cysts, lesions that are infiltrative or inflammatory, and those that are vascular or metastatic. Diagnosing sarcoidosis typically involves a combination of clinical evaluation, medical history, and multiple diagnostic tests. There are numerous proposed diagnostic criteria. The histological finding of non-caseating granulomata remains the hallmark of the disease. However, a biopsy is not always practical or sufficiently safe. Neurosarcoidosis diagnosis primarily depends on factors beyond histology such as cerebrospinal fluid results or magnetic resonance imaging (MRI) [7-8]. It is common to find abnormalities in the cerebrospinal fluid (CSF) of individuals with neurosarcoidosis. Over 80% of cases exhibit these abnormalities upon initial presentation. The most frequent abnormality observed is an elevation in protein levels, which can sometimes reach very high levels and indicate a dysfunction in the blood-brain barrier. Additionally, 55% of patients show lymphocytosis in their CSF [8]. MRI is very sensitive in detecting abnormalities in neurosarcoidosis, but it is nonspecific. In particular, It is common to observe periventricular T2-hyperintense lesions that can resemble multiple sclerosis features and impact the corpus callosum [9]. It is worth noting that about half of the patients with hypothalamic-pituitary sarcoidosis may appear normal in radiological tests [10]. Prompt diagnosis and initiation of corticosteroids and immunosuppressants can be lifesaving for controlling the inflammatory aspects of the disease. However, when neurosarcoidosis affects the endocrine system, these treatments may not fully restore compromised pituitary hormones to normal levels. Consequently, hormone replacement therapies become inevitable.

## Conclusions

Panhypopituitarism secondary to neurosarcoidosis is a rare presentation. It occurs due to the infiltration of the pituitary gland or the infiltration of the hypothalamus affecting the hypothalamic-pituitary axis. Infiltrative disorders, such as sarcoidosis, should be considered a differential when evaluating patients with symptoms consistent with panhypopituitarism. Prompt diagnosis and initiation of corticosteroids and deficient hormones can be lifesaving.
